# Developmentally Tailored Telehealth-Delivered Cognitive-Behavioural Therapy for Adolescents with Body Dysmorphic Disorder: A Multiple Baseline Design

**DOI:** 10.1007/s10802-026-01452-z

**Published:** 2026-04-18

**Authors:** Cassie H. Lavell, Ella L. Oar, Ronald M. Rapee

**Affiliations:** Lifespan Health and Wellbeing Research Centre, Macquarie University, Sydney, Australia

**Keywords:** Body dysmorphic disorder, Adolescents, Cognitive-behavioural therapy, Telehealth

## Abstract

There is a paucity of research on the treatment of adolescent body dysmorphic disorder (BDD). Cognitive-behavioural therapy (CBT) has shown initial promise, yet many adolescents do not recover and face significant barriers in accessing treatment. The current study examined the acceptability and preliminary efficacy of developmentally tailored telehealth-delivered CBT for adolescents with BDD, using a single-case, nonconcurrent, multiple baseline design. Recruited from a broader study of correlates of BDD, 22 adolescents were eligible and were offered treatment. Sixteen (12 to 16 years; *n* = 5 boys) enrolled and were randomised to a one-, two- or three-week baseline period. Primary outcomes, assessed at post-treatment and again at 2-month follow-up, included BDD diagnostic status and symptom severity. Secondary outcomes included insight, functional impairment, comorbidity, anxiety, and depression. Acceptability and feasibility were examined by measuring participants’ treatment satisfaction, as well as examining drop-out rates. Seven participants dropped out of the study early, while 8 completed the 12-session treatment, and 1 completed 9 sessions. Among the treated sample, treatment satisfaction was high. BDD severity remained stable throughout the baseline and tended to improve following CBT. At post-treatment, 66.7% of the treated sample (37.5% of the total enrolled) participants were treatment responders, and 55.6% (31.3% enrolled) were still responders at 2-month follow-up. Significant reductions on most secondary outcomes were also observed. However, most participants remained symptomatic following treatment, and many dropped out early in treatment. Future studies are much needed to improve outcomes for adolescents with BDD.

## Introduction

Body dysmorphic disorder (BDD) affects approximately 1 to 2% of the population (Veale et al., 2016). Classified as an obsessive-compulsive related disorder (OCRD) in the Diagnostic and Statistical Manual for Mental Disorders, 5th Edition Text Revision (DSM-5-TR; American Psychiatric Association, 2022), BDD is characterised by a preoccupation with perceived flaws in physical appearance and is associated with significant distress and impairment in functioning (Phillips et al. 2006; Rautio et al. 2022a, b). Without treatment, BDD tends to be chronic and unremitting (Phillips et al. 2013a, b).

BDD typically begins during adolescence and affects youth at comparable prevalence rates to adults (1–2%; Krebs et al., 2025; Schneider et al., 2017). An earlier onset of BDD is associated with the development of more severe symptoms (Bjornsson et al., 2013). Adolescents with BDD, compared to adults, show significantly higher lifetime rates of suicide attempts and a greater conviction that their appearance-related beliefs are accurate (known in the literature as “poor insight”; Phillips et al., 2006). Despite the severity and impact of BDD in youth, research into treatments for this population is only just emerging. In adults, there is evidence to suggest cognitive-behavioural therapy (CBT) and selective serotonin reuptake inhibitors (SSRI) may be efficacious treatments for BDD (Liu et al., 2024; Williams et al., 2006). Adult CBT protocols typically involve psychoeducation, cognitive restructuring, exposure with response prevention (ERP), relapse prevention, and some form of perceptual retraining. Perceptual retraining techniques (e.g., mirror/attention training) teach the individual with BDD how to attend to their appearance more globally and nonjudgmentally.

## Effectiveness of CBT

In adolescents with BDD, some preliminary support for the utility of CBT has been found in a number of single case studies (Sobanski & Schmidt, 2000), case series’ (Greenberg et al., 2016; Krebs et al., 2012), and a large uncontrolled study of flexibly-delivered CBT combined with medication in 140 young people (aged 10 to 18 years; Rautio et al. 2022a, b). However, to date, there is only one published randomised controlled trial (RCT) testing CBT in 30 adolescents with BDD (Mataix-Cols et al., 2015), with a protocol modelled on CBT for adults with BDD and adolescents with obsessive-compulsive disorder (OCD). The treatment involved 14 sessions of CBT (total of 15 h) including psychoeducation, goal setting, exposure with response prevention (ERP), and relapse prevention. Parents were involved in treatment at differing degrees depending on the individual formulation, such as the level of parental involvement in BDD-related rituals. In some cases, participants received additional modules such as mirror retraining (66.7% of the sample) and attention training (53.3%).

Mataix-Cols et al. (2015) found that CBT was superior to the control condition with all 15 participants in the CBT condition completing treatment and follow-up assessments. 40% were classified as responders at post-treatment and 2-month follow-up, with treatment response defined by at least a 30% reduction in BDD symptoms on the adolescent version of the Yale-Brown Obsessive-Compulsive Scale Modified for BDD (BDD-YBOCS-A). In a 12-month follow-up study, 50% were classified as responders, however, the majority of adolescents remained symptomatic, two patients attempted suicide, and 27% still wanted cosmetic surgery after CBT (Krebs et al., 2017). Therefore, while CBT may be effective for many young people with BDD, the evidence-base is limited, and there is a need to improve interventions to increase efficacy. The greater severity of symptoms and lower insight in youth suggests this age group may require adapted and developmentally tailored treatments, relative to adults.

## Adolescent Developmental Factors

While comprehensive models of BDD have been developed which cover both biological and environmental risk factors (Neziroglu et al., 2008; Wilhelm et al., 2012), adult CBT treatments have focused specifically on proximal maintaining factors such as selective attention to perceived flaws, overvaluation of physical appearance, behavioural avoidance, and safety behaviours. Most studies that have tested these proposed models have used adult samples, with a scarcity of research in youth. Adolescents with BDD may require adapted treatment approaches that are better tailored to their developmental needs.

Peer relationships and the social context may be particularly salient for adolescents with BDD. From the limited cross-sectional research to date, findings suggest that there may be a relationship between peer victimisation and BDD in adolescents. Two studies have found that children and adolescents with BDD report higher rates of peer victimisation relative to those without BDD (Monzani et al., 2024; Neziroglu et al., 2018). Although more frequent appearance-based peer victimisation (i.e., being teased about looks) was not found in adolescents with BDD relative to control groups in another study (Lavell et al. 2024b), this study did find that adolescents with BDD experienced significantly more distress from appearance-based victimisation than controls. Moreover, the same study also found that adolescents with BDD perceived significantly less social support from their friends relative to non-clinical controls. The cross-sectional nature of these studies means causal relationships cannot be determined. However, it is possible that BDD symptoms are perpetuated by peer relationship problems. For example, peer difficulties may lead to increased feelings of rejection and in turn, attempts to avoid further rejection through social avoidance or camouflaging perceived flaws.

The role of social media may also be an important consideration for the treatment of adolescent BDD, given the relationship between online appearance comparisons and body dissatisfaction in young people (Fardouly & Vartanian, 2016). Emerging research in non-clinical samples has found a cross-sectional association between the frequency of image-based, but not text-based, social media use and body dysmorphic symptoms in adolescents and adults (Alsaidan et al., 2020; Gupta et al., 2023). Moreover, in their study of 209 adolescents (16 to 18 years old), Gupta et al. (2023) also found that appearance-based motivations for social media use was uniquely associated with body dysmorphic symptoms. In a clinical study, adolescents diagnosed with BDD were found to report making more frequent online appearance comparisons when using social media than adolescents with anxiety disorders and non-clinical controls (Lavell et al. 2024b), suggesting this may be a social media behaviour more heightened in young people with BDD. It is possible that image-based social media use and online appearance comparisons may perpetuate adolescents’ appearance distress over time, via exposure to idealised images and an increased focus on appearance. Therefore, developing more adaptive social media behaviours may be a useful treatment target to enhance current CBT protocols for adolescents with BDD.

In addition to social factors, there is evidence to suggest parenting variables may be associated with BDD in adolescents. For example, there is emerging evidence to suggest that, similar to a process observed in paediatric OCD and anxiety disorders (Lebowitz et al., 2016), parents of adolescents with BDD frequently become involved in compulsive symptomatology (e.g., by providing reassurance, buying beauty products, altering family routines; Jassi et al., 2020; Lavell et al., 2025). This family accommodation may maintain BDD symptomatology, whereby parents help their child temporarily avoid distress, but inadvertently prevent opportunities for new learning and coping. Research thus far suggests that parental accommodation of appearance concerns is the norm within families of adolescents with BDD, with between 92% and 100% of parents reporting they engage in accommodation (Hogg et al., 2024; Lavell et al., 2025). Hence, addressing this process in treatment is likely to be important in the majority of cases of adolescent BDD.

There may also be important cognitive factors to consider for adolescents with BDD. For example, adolescents with BDD have been found to have lower insight compared to their adult counterparts (Phillips et al., 2006) and adolescents with other mental disorders (Lavell et al. 2024a). There is also evidence that adolescents with BDD are more likely to experience executive functioning difficulties than non-clinical controls, including those involved in shifting and cognitive flexibility (Lavell et al. 2024a; Rajabi et al. 2022). Given the low insight and cognitive inflexibility observed in adolescents with BDD, motivational interviewing and attention training may need to be standard components of treatment.

## Treatment Accessibility

Despite emerging evidence for the efficacy of CBT for treating adolescent BDD, research suggests that less than half of adolescents with the disorder receive psychotherapy for their appearance concerns (Krebs et al., 2023). There are many treatment barriers for individuals with BDD including limited access to therapists trained in assessing and treating BDD (McCausland et al., 2021). Hence, efforts to improve treatment accessibility are much needed. Remote telehealth delivery of CBT, whereby a therapist delivers the intervention to the participant via videoconference, may help to overcome accessibility barriers. Evidence for the effectiveness of telehealth CBT for treating BDD is limited, with only one study thus far examining telephone delivery of CBT in a small case series with adult participants (Drüge et al., 2022). While telehealth CBT has been shown to be effective in treating youth with OCD (Hollmann et al., 2022), the effectiveness and acceptability of telehealth CBT for adolescents with BDD has not yet been examined. The development of effective telehealth treatments would help to overcome the accessibility barriers experienced by adolescents with BDD and their families.

## Study Aims

The current study aimed to test the preliminary efficacy, feasibility, and acceptability of a developmentally tailored, telehealth-delivered, CBT approach to treating BDD in adolescents. Modifications were made to current treatment protocols, including videoconference delivery, increased and standardised family involvement in therapy and additional modules targeting peer relationships and social media use. Motivational interviewing and attention training were also included as standard components of the treatment. Acceptability and feasibility were evaluated by examining participant retention, treatment satisfaction, and homework compliance. The efficacy of the treatment was evaluated using a single-case non-concurrent multiple-baseline case series design. Participants were randomly assigned to either a one-week, two-week or three-week baseline period. It was hypothesised that BDD symptom severity would remain stable across the baseline period, and then significantly improve following treatment. Moreover, it was predicted that significant reductions would be observed from pre- to post-treatment, and at 2-month follow-up, in BDD severity, diagnostic status, clinician-rated global severity, insight, functional impairment, anxiety, and depression. Finally, it was also expected that the treatment approach would be deemed acceptable to adolescents and their parents. The study also aimed to explore whether there were changes in the pattern of comorbidity following treatment.

## Method

### Participants

Participants were recruited from a larger study examining correlates of BDD in adolescents (including peer relationship and family factors) run via the Macquarie University School of Psychological Sciences. This study was advertised to mental health professionals and through school newsletters. Details of the larger study, including its sample, are reported in Lavell et al. (2024b). Participants were deemed eligible for the current treatment study if they were (a) between the ages of 12 to 17 years, (b) experiencing a primary DSM-5-TR diagnosis of BDD, (c) able to be supported by at least one parent to attend all assessment and treatment appointments and (d) if on psychotropic medication, the dose was stable for at least eight weeks. Exclusion criteria for the study were (a) intellectual disability, (b) experiencing difficulties of a higher priority or requiring immediate action (e.g., suicidal intent or plan, current abuse/neglect, or unmanaged psychotic symptoms), (c) body image concerns better accounted for by an eating disorder, (d) undergoing concurrent psychotherapy or not able to remain on a stable dose of medication. Families were screened for eligibility following their participation in the larger study of the nature of BDD, and all eligible participants were offered treatment until the sample size was reached.

Initial assessments and enrolment took place between July 2021 and December 2022. In total, 22 participants from the broader study were eligible and offered treatment. Five did not wish to participate as they were pursuing psychological therapy elsewhere, and one adolescent did not wish to engage in a psychological therapy. A total of 16 participants enrolled in the treatment study and were subsequently randomised to one of three baseline conditions.

### Design

The treatment was evaluated using a single-case, non-concurrent multiple baseline design. Single-case designs are endorsed by the evidence-based treatment movement (Chambless, 1995), as they allow for the initial evaluation of the efficacy of interventions in a controlled manner (Jarrett & Ollendick, 2012). Single-case designs are often preferable for determining the preliminary feasibility of treatments, due to their time- and cost-effectiveness relative to larger RCTs (Horner et al., 2005). While non-concurrent designs have been criticised for potential threats to internal validity, several researchers have argued they have equivalent experimental control to concurrent designs and have benefits such as ecological validity (Kratochwill et al., 2022; Slocum et al., 2022). Hence, a non-concurrent design was selected to replicate the conditions of community clinics, in which young people present sequentially (Ollendick et al., 2021). Participants were randomly assigned to one of three baseline periods: one-week (*n* = 5), two-weeks (*n* = 6), or three-weeks (*n* = 5), using a computer-generated (www.randomization.com) list of randomly permuted blocks. Participants received their randomization number consecutively, based on the order of their enrolment in the study.

### Measures

#### Primary Outcomes

The BDD Diagnostic Module (Phillips, 2005, 2017), a semi-structured diagnostic interview, was used to assess DSM-5-TR criteria for BDD. As part of our broader study of adolescent BDD that included 26 participants with BDD as well as 52 controls, 15% of BDD Diagnostic Module interviews were reviewed for interrater reliability, and there was 100% agreement on BDD diagnostic status between raters.

DSM-5-TR comorbidity was determined using the Mini International Neuropsychiatric Interview for Children and Adolescents – Child Version (MINI-Kid; Sheehan et al., 2010). The MINI-Kid is a structured diagnostic interview that assesses a range of mental disorders and neurodevelopmental conditions in youth based on DSM-5-TR criteria, and also includes a 7-item screener for Autism. Based on this screener, Autism is deemed either not present or “cannot be ruled out” if at least one item is endorsed. The interview has demonstrated substantial interrater and test-retest reliability, as well as high sensitivity and specificity (Sheehan et al., 2010). Both diagnostic interviews were delivered via videoconference (Zoom), a method that has demonstrated comparable reliability and validity to face-to-face interviews (Elford et al., 2000; Lyneham & Rapee, 2005).

The Body Dysmorphic Disorder Modification of the Yale-Brown Obsessive-Compulsive Scale for Adolescents (BDD-YBOCS-A; Phillips et al., 1997) was used as the primary outcome measure of BDD severity. The scale is a 12-item semi-structured clinician-administered interview measuring time, interference, distress, resistance, and control over BDD thoughts and behaviours (e.g., “*How much time do you spend thinking about this problem with how you look?*”). It also includes items assessing insight and avoidance. Scores range from 0 to 48. The BDD-YBOCS-A has good psychometric properties and is sensitive to changes in BDD severity (Monzani et al., 2023). In the current study, reliability for the BDD-YBOCS-A at pre-treatment was *α* = 0.95.

#### Baseline and Weekly Monitoring

 An adolescent-report, 10-item version of the BDD-YBOCS (BDD-YBOCS-SR) was used to measure BDD severity across pre-treatment, baseline, during treatment, post-treatment, and follow-up. Items are consistent with the clinician-administered version of the BDD-YBOCS, except items for avoidance and insight are removed. Items are scored on a 5-point scale, with total scores ranging from 0 to 40. This measure has demonstrated comparable psychometric properties to the 12-item clinician-administered version (Phillips et al., 1997; Marques et al., 2011). Reliability in the current study at pre-treatment was *α* = 0.94.

#### Secondary Outcomes

Parents also completed a 10-item version of the BDD-YBOCS modified for parents (BDD-YBOCS-PR) at pre-treatment, post-treatment, and follow-up. Items of the BDD-YBOCS-SR were modified to suit a parent-report format, for example, “*How much time do you spend thinking about your looks in a day*?” became “*How much time does your child spend thinking about their looks in a day*?”. In the current study, Cronbach’s alpha was *α* = 0.86 at pre-treatment.

The Clinician Global Impressions severity (CGI-S) and improvement (CGI-I) scales (Busner & Targum, 2007) were used to measure clinician-reported BDD severity and improvement. Each scale is rated on a 7-point scale (1 to 7). These scales are widely used in treatment trials, including the first RCT for adolescent BDD (Mataix-Cols et al., 2015).

The Brown Assessment of Beliefs Scale (BABS; Eisen et al., 1998), a 7-item semi-structured clinician-administered measure, was used to assess participants’ insight. Total scores range from 0 to 24, and higher scores indicate “poorer” insight. The BABS has been found to have good psychometric properties in samples of adults with BDD (Phillips et al. 2013a, b). It has also been utilised with adolescents with BDD (Mataix-Cols et al., 2015). In the current study, Cronbach’s alpha was *α* = 0.90 at pre-treatment.

To measure BDD-related functional impairment, an adapted version of the parent and child versions Child Anxiety Life Interference Scale (CALIS; Lyneham et al., 2013) was used. For the current study, CALIS items were modified to reflect interference due to appearance concerns. For example, the statement “*How much do fears and worries make it difficult for you to do the following things?*” was changed to “*How much does feeling upset about the way you look make it difficult for you to…*” The parent scale is 16 items (range = 0 to 64), and the child scale is 9 items (range = 0 to 36). Reliability was *α* = 0.79 for the parent version *α* = 0.89 for the adolescent version.

An abbreviated 18-item parent‐ and child‐report version of the Spence Children’s Anxiety Scale (SCAS; Spence, 1998), comprising the generalised anxiety, social anxiety and separation anxiety subscales, was used to assess participants’ anxiety (range = 0 to 54). Reliability was high at pre-treatment for parent report (*α* = 0.91) and adolescent report (*α* = 0.94).

The parent and child versions of the 13-item Short Mood and Feelings Questionnaire (SMFQ; Angold et al., 1995) were used to measure participants’ depressive symptoms. Items are rated on a 3-point scale (0 = *Not True*, 2 = *Always True*). Total scores ranged from 0 to 26 with higher scores indicating more depressive symptoms. In the current study, reliability was *α* = 0.86 for the parent version and *α* = 0.91 for the adolescent version.

#### Treatment Satisfaction

One-week post-treatment, adolescents’ and parents’ treatment satisfaction was measured using adapted versions of the Child Treatment Satisfaction (CTS) and Parent Treatment Satisfaction (PTS) scales developed by Ollendick et al. (2015). Each scale consists of 6 items (e.g., “*Treatment helped me cope better with my appearance worries*”) rated on a 5-point scale (1 = *Strongly Disagree*, 5 = *Strongly Agree*), with higher scores indicating greater treatment satisfaction (range = 6 to 30). Parents were also asked about the length of treatment, i.e., whether treatment was “just the right length”, “too long” or “too short”. Additionally, adolescents and their parents were asked to rank each treatment component from most important to least important in helping them overcome BDD. Reliability for the 6-item scale in the current study was *α* = 0.91 for the CTS and *α* = 0.80 for the PTS.

#### Homework Compliance

 Participants’ homework compliance was measured using a clinician-rated 7-point scale (Park et al., 2014) from 0 (*Did not complete any assigned homework*) to 6 (*Completed all homework and made efforts above and beyond assignments*), completed at sessions 2 to 12. Participants’ mean score across the 11 sessions was used as the total score for homework compliance (range = 0 to 6).

### Procedure

#### Pre-Treatment and Baseline

 The study protocol was approved by the Macquarie University Human Research Ethics Committee and the trial was registered with the Australian New Zealand Clinical Trials Registry (ACTRN12622000409774). Predominantly, parents referred their child to the study, but *n* = 3 adolescents (aged 16–17) self-referred via email. An initial telephone call was conducted with the parent to screen for eligibility for the larger study of BDD. During this call, parents were asked about their child’s history of psychiatric or neurodevelopmental diagnoses. Following the screening call, diagnostic interviews were administered by the first author, a registered clinical psychologist, with the adolescent via Zoom. Once participants were deemed eligible for the larger study based on the interview, they were offered the current treatment study and their diagnostic data collected for the broader study was used as pre-treatment data in the current study. Next, they completed informed consent forms and pre-treatment online questionnaires via LimeSurvey and were randomised to a baseline period of either 1-week, 2-weeks, or 3-weeks. During the baseline period, adolescents were emailed the BDD-YBOCS-SR to complete each week.

#### Intervention

Following the baseline period, participants attended twelve, 90-minute sessions (18 h) of CBT delivered via telehealth (Zoom). A 90-minute session duration was chosen to allow enough time for adequate skill practice, particularly for ERP. This 90-minute approach has been shown to be effective in a previous case study of an adolescent with BDD (Aldea et al., 2009). Consistent with existing adult and adolescent CBT treatment manuals for BDD (Mataix-Cols et al., 2015; Veale & Neziroglu, 2010; Phillips, 2005; Wilhelm et al., 2012), psychoeducation, cognitive restructuring, and ERP were core components of the treatment protocol. However, several adaptations were made to ensure content was developmentally tailored to maximise treatment outcomes. Adaptations were made based on CBT formulations of BDD (e.g., Wilhelm et al., 2012) and the adolescent BDD literature outlined within the introduction. These included increased parent involvement (20–30 min per session in addition to a 90-minute family session), motivational interviewing and attention/mirror retraining provided to all participants to address cognitive inflexibility and selective attention, and skills training to support the development of healthy peer relationships and social media use. The 12-session protocol involved the following components (summarised in Table 1):

**Table 1:** Summary of treatment content

Session	Topics Covered
Session 1	Psychoeducation Motivational interviewing*
Session 2	Cognitive restructuring
Session 3	Exposure with response prevention
Session 4	Exposure with response prevention Perceptual and attention training*
Session 5	Exposure with response prevention
Sessions 6–7	Exposure with response prevention Family problem solving * Addressing family accommodation*
Sessions 8–9	Exposure with response prevention Peer relationship and social media skills*
Session 10–11	Exposure with response prevention
Session 12	Relapse prevention

* Modified and/or additional treatment components

##### Psychoeducation 

In the first treatment session, adolescents and their parents received psychoeducation about BDD, including how to differentiate typical appearance concerns from BDD, risk factors involved in the development of BDD (e.g., neurobiological factors and environmental factors), and the maintenance cycle of BDD. Motivational interviewing was conducted for all participants, given the sample’s overall low insight, by identifying the costs versus benefits of continuing to perform compulsive appearance-related behaviours and developing an alternative belief to challenge their current BDD beliefs (i.e., the Theory A versus Theory B technique; Veale & Neziroglu, 2010). The therapist and participant also developed an individualised CBT formulation, and from this, a rationale for treatment, including ERP was provided. Participants also set goals they hoped to achieve by the end of the treatment program.

##### Cognitive Restructuring 

In the second treatment session, participants were provided education about the thought-feeling-behaviour connection. Cognitive distortions (“unrealistic thoughts”) were explained and discussed in relation to the young person’s appearance-related beliefs. The cognitive restructuring process was explained and practised during the session. This involved identifying appearance-related cognitions and inflated beliefs about the importance of appearance (e.g., “*If I do not look good*,* I will never make friends*”), exploring the evidence for and against, and identifying more realistic thoughts. Participants were encouraged to practice cognitive restructuring as homework. Cognitive restructuring continued throughout future sessions when needed.

##### Exposure with Response Prevention

 Sessions 3 to 11 primarily focussed on ERP. Exposure hierarchies were initially developed, and steps were practised in-session with the young person. ERP was conducted using an inhibitory learning approach (Craske et al., 2014), whereby tasks were designed to maximally violate expectancies. Typically, ERP was conducted within the home initially, and in later sessions ERP was conducted in public places with therapist guidance over Zoom or telephone (e.g., resist safety behaviours when walking through a local shopping centre). ERP was adapted to suit the telehealth format. For example, several participants were highly anxious about being viewed on video camera or viewing themselves on the screen. Therefore, initial accommodations were made to increase engagement (e.g., turning off the camera or self-view), but ERP was later used to address these fears. Weekly ERP goals were set for homework, and homework was reviewed at the beginning of each session. Families were encouraged to use rewards to enhance motivation to complete ERP homework.

##### Perceptual and Attention Training

During session 4, perceptual and attention training skills were delivered to supplement ERP, based on adult BDD treatment protocols (Phillips, 2005; Veale & Neziroglu, 2010; Wilhelm et al., 2012). Education was first provided to the young person on the role of attention in maintaining BDD, and how cognitive inflexibility might prevent them from easily shifting their attention away from detail-oriented processing of their appearance. Mirror retraining was modelled by the therapist, then practised by the participant, whereby they were encouraged to stand arm’s length from the mirror and shift their attention from perceived flaws to viewing their overall appearance, describing themselves in objective and non-judgmental language. Attention training was also practised to reduce self-focussed attention (e.g., when out in public, when at school, with friends), requiring the participant to pay attention externally to their five senses. Both mirror retraining and attention training exercises were set as daily homework practice and were re-visited in later sessions to assist with ERP.

##### Family Support

Session 6 focussed on parent involvement in BDD. Both parents were invited to attend, but in cases where only one parent attended, it was encouraged that information was passed on to the other parent. Families were given education on the role of family accommodation in maintaining BDD. Problem-solving was used to identify solutions to common family concerns (e.g., how to react to appearance-based criticism and comments from extended family members). ERP hierarchies were developed specifically to address family accommodation. Parents were taught skills to provide their child empathy, support, and encouragement when they experienced BDD-related distress. The session was delivered flexibly, whereby all topics were covered for all participants, however certain topics were covered in more detail if more important for the family (e.g., more time was spent on providing empathy and support with families who were identified as struggling with this). ERP homework for reducing accommodation was set, and this was reviewed during session 7.

##### Peer Relationship Skills

Session 8 primarily focussed on education and skills training for various social situations related to BDD including peer victimisation, poor social support, “appearance talk” with friends, and social media use (e.g., upward comparisons, viewing idealised images). In all cases, all topics were covered, however more time was spent on topics the adolescent identified they were most affected by. Problem-solving and assertiveness skills were rehearsed to assist with any difficult social situations experienced by the young person. For participants who identified social media as a maintenance factor for their BDD, coping strategies were explored such as scheduling a “social media detox” and/or unfollowing accounts with idealised images. For all participants, ERP was emphasised, and hierarchies were developed to reduce avoidance and/or safety behaviours used in social situations. Homework typically involved conducting ERP in social situations and practising other skills such as problem-solving, assertiveness and changing social media behaviours. These skills were rehearsed again in session 9.

##### Relapse Prevention

 The final treatment session involved a review of progress, celebration of goals achieved, identification of which strategies were most helpful, and planning for overcoming any remaining BDD symptoms and/or future fears.

#### Post-Treatment Assessments

 One-week post-treatment, and again two-months post-treatment, independent assessors completed diagnostic interviews via Zoom. Independent assessors were post-graduate trained psychologists experienced in administering diagnostic interviews for adolescent emotional disorders. Pre-treatment severity was masked, however the assessment time point was unmasked, as the interviewers assessed for protocol deviations (e.g., other treatments) between post-treatment and two-months follow-up. Post-treatment assessments (BDD-YBOCS-A, BABS, CGI-S and CGI-I) were reviewed and moderated in consensus meetings with the second author. Adolescents and parents completed post-treatment online questionnaires via Limesurvey at each time point. Treatment satisfaction scales were completed at one-week post-treatment. Families who completed all post-treatment assessments were given a $30 gift voucher.

#### Treatment Adherence 

The therapist for all treatment sessions was a registered clinical psychologist (the first author) with experience delivering CBT to treat OCRDs, including BDD, in adolescents. The current study opted for a pragmatic and cost-effective approach to rating treatment adherence. Following each treatment session, the therapist completed an 8-item treatment adherence questionnaire assessing how well they adhered to each component of the treatment manual (e.g., “*Performed an in-session exposure task with the client*”) on a 7-point scale (0 = Not at all, 6 = Excellent). Mean treatment adherence scores ranged between “Good” and “Excellent”, i.e., from 4.44 (session 8) to 5.75 (session 12).

### Data Analysis

Visual inspection of the BDD-YBOCS-SR case series data was used to determine stability of the baseline, as well as non-parametric Wilcoxon Signed Rank pairwise tests for the 1-week baseline group, and Friedman tests for the 2-week and 3-week baseline groups. Given the small sample size, non-parametric Friedman tests followed by post-hoc Wilcoxon Signed Rank tests were conducted to examine participant changes over time (from pre-treatment to 1-week and 2-months follow-up) on primary and secondary outcome measures. Effect sizes (*r*) for Wilcoxon Signed Rank tests were calculated by dividing the *z* statistic by square root of the sample size. Participants were considered treatment responders if they had a greater than 30% reduction on the BDD-YBOCS-A, and in remission if their BDD-YBOCS-A score was less than or equal to 16. Response and remission definitions are based on a prior study of 153 adults with BDD (Fernández de la Cruz et al., 2021). *P*-values were not adjusted for multiple comparisons as the study was exploratory in nature and utilised single-case design. Last observation carried forward (LOCF) was used to manage missing data at post-treatment and two-month follow-up.

## Results

### Participant Characteristics

Participants were aged 12 to 16 years (*M*_*ag*e_ = 14.19, *SD* = 1.27). Five participants (31%) were boys, one of whom was transgender. The majority (*n* = 13) were White; 1 was Asian; 1 was Aboriginal; and 1 was European. Two participants had previous Autism diagnoses from other health professionals, and *n* = 3 had a previous ADHD diagnosis. On average, participants had been experiencing BDD symptoms for 3.50 years (*SD* = 1.67, range = 1–7 years). 81% of participants had at least a secondary DSM-5-TR diagnosis based on their MINI-Kid interviews, 56% had a tertiary diagnosis, and 31% had four or more diagnoses. Clinical characteristics are presented in Table 2.

**Table 2:** Participant pre-treatment clinical characteristics

	*N* = 16	
	*n*	%
Poor or Delusional Insight	10	62.5%
History of Self-Harm	9	56.3%
History of Suicidal Ideation	9	56.3%
Current SSRI	4	25.0%
Past Psychological Treatment for BDD	1	6.3%
Primary Body Area of Preoccupation		
Legs	5	31.3%
Face shape	4	25.0%
Skin	2	12.5%
Stomach	2	12.5%
Other (nose, muscle size, shoulders)	3	18.75%
MINI-Kid Comorbidity		
Social Anxiety Disorder	7	43.8%
Major Depressive Disorder	6	37.5%
Generalised Anxiety Disorder	4	25.0%
Attention-Deficit/Hyperactivity Disorder	4	25.0%
Specific Phobia	3	18.8%
Obsessive-Compulsive Disorder	2	12.5%
Panic Disorder	1	6.3%
Separation Anxiety Disorder	1	6.3%
Post-Traumatic Stress Disorder	1	6.3%
Oppositional Defiance Disorder	1	6.3%
Chronic Tic Disorder	1	6.3%
Binge Eating Disorder	1	6.3%
Autism Not Ruled Out	15	93.8%

Note: *SSRI* = selective serotonin reuptake inhibitor

### Participant Retention

Of the 16 participants, one participant was unable to schedule appointments so did not proceed after randomisation. Six participants dropped out early in treatment (i.e., by session 5). Reasons for dropout included parent disengagement (*n* = 1), adolescent unwilling to continue (*n* = 2), symptoms improved in the early stages of treatment (*n* = 1) and other issues became the primary concern (*n* = 2). Of the adolescents who were unwilling to continue the treatment, one wished to pursue a rhinoplasty instead of therapy, and one stated they no longer wanted to talk about their BDD. No significant differences in baseline characteristics were found between participants who dropped out following randomisation (*n* = 7) and those who continued in treatment (*n* = 9), including BDD-YBOCS severity, *t*(14) = -0.45, *p* = .663, insight, *t*(14) = -0.78, *p* = .451, or number of comorbid disorders, *t*(14) = -1.46, *p* = .166.

Eight of the 9 remaining participants completed the entire 12-session treatment. The 12 sessions were completed over an average of 16 weeks (*SD* = 2.26; range = 12 to 19 weeks). Session delays were typically due to illness or holidays. For four participants, sessions were occasionally delayed as the participant refused to attend the session. When this occurred, the therapist checked in briefly with the parent (< 10 min) and the appointment was rescheduled for the following week. The other participant attended 9 treatment sessions of the 12, declining the final sessions due to no longer wanting to discuss their BDD. They did not attend sessions 10 and 11 which involve continued ERP and session 12 which focuses on relapse prevention. This participant declined to participate in post-treatment diagnostic assessments, however, they did complete post-treatment questionnaires 1-week after they would have been due to complete their final (12th ) session. One parent did not complete the BDD-YBOCS-PR at pre-treatment, so data for this outcome variable was conducted on *n* = 15 participants.

### Protocol Deviations

One participant commenced stimulant medication for ADHD mid-way through treatment (between sessions 5 and 6). Five participants commenced other treatments between the post-treatment and 2-month follow-up assessment (*n* = 3 saw a psychologist for treatment of other psychological problems; *n* = 1 commenced an SSRI; *n* = 1 commenced an SSRI and saw a psychologist for one session).

### Treatment Satisfaction

Among participants who continued in treatment (*n* = 9), treatment satisfaction scores were high for adolescents (*M* = 24.78, *SD* = 3.93, range = 19–30) and parents (*M* = 25.22, *SD* = 2.28, range = 23–30). All parents and most adolescents (88.9%) either agreed or strongly agreed that treatment was helpful. Over half (55.6%) of parents thought the 12-session treatment was “just the right length”, while 22.2% thought it was too long, and 22.2% thought it was too short. For adolescents, 66.7% ranked realistic thinking in their top 3 most important treatment components. Moreover, 44.4% ranked mirror/attention training and ERP in their top 3. In contrast, parents most frequently ranked psychoeducation about BDD (77.8%), support of the therapist (66.7%) and realistic thinking (66.7%) in their top 3 most important treatment components.

### Homework Compliance

Overall, the treated sample’s (*n* = 9) mean homework compliance score fell between the range of “*Completed some of assigned homework but less than 50%*” and “*Completed approximately 50% of assigned homework*” (*M* = 2.85, *SD* = 0.67; range = 1.67–3.91).

### Single-Case Baseline Data

**Fig. 1:**
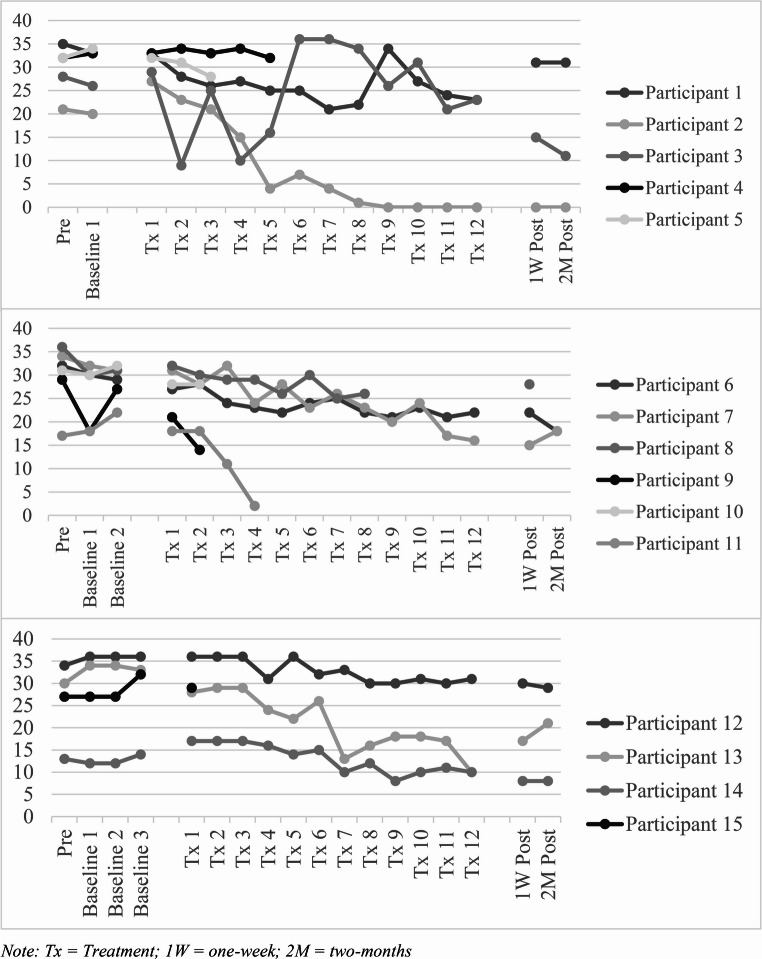
BDD-YBOCS-SR scores at baseline, treatment and post-treatment

Visual inspection of single-case data (see Fig. 1) for *n* = 15 participants who completed the baseline, demonstrated that BDD-YBOCS-SR scores remained relatively stable across the baseline period and, in most cases, reduced following treatment. Non-parametric Wilcoxon and Friedman tests also showed stability of the baseline, as there were no significant differences BDD-YBOCS-SR across the 1-week baseline (*N* = 5; *z* = -0.14, *p* = .888, *r* = .06), 2-week baseline (χ^2^(2, *N =* 6) = 3.00, *p* = .223), or 3-week baseline (χ^2^(3, *N =* 4) = 3.60, *p* = .308).

### Primary Outcomes

#### BDD Severity

 Friedman tests, reported in Table 3, demonstrated significant changes in BDD-YBOCS-A severity across the three time points among those who continued in treatment. Pairwise Wilcoxon tests found significant reductions in BDD-YBOCS-A severity with large effects from pre-treatment to post-treatment (*z* = -2.38, *p* = .017, *r* = .80) and from pre-treatment to 2-month follow-up (*z* = -2.52, *p* = .012, *r* = .84). There were no significant differences between post-treatment and 2-month follow-up BDD-YBOCS-A scores (*z* = -0.77, *p* = .444, *r* = .26). As shown in Table 3, this pattern of results remained the same when early dropouts were included in the analyses, with LOCF used to handle missing data.

In the treated sample (*n* = 9), there was a significant negative correlation between BDD-YBOCS-A severity at baseline and amount of change on the BDD-YBOCS-A at post-treatment (*r* = -72, *p* = .029) and follow-up (*r* = − .71, *p* = .031). There was also a significant negative correlation between baseline insight and BDD-YBOCS-A change at post-treatment (*r* = − .81, *p* = .009), but the relationship was non-significant at 2-month follow-up (*r* = − .64, *p* = .066).

#### Responders and Remitters

 On the BDD-YBOCS-A, *n* = 6 participants were classified as responders at post-treatment, which was 66.7% of the treated sample and 37.5% of the enrolled sample. Two participants were in remission at post-treatment (22.2% of treated; 12.5% of enrolled). At the 2-month follow-up, *n* = 5 were responders (55.6% of treated; 31.3% of enrolled) and *n* = 3 were in remission (33.3% of treated; 18.8% of enrolled). Two participants no longer met DSM-5-TR criteria for BDD at post-treatment and *n* = 3 no longer met criteria at 2-month follow-up based on the BDD Diagnostic Module, while the other participants retained their diagnosis of BDD at both time points.

### Secondary Outcomes

Friedman tests for secondary outcomes are reported within Table 3.

#### Self- and Parent-Report BDD Severity

Wilcoxon pairwise tests showed significant improvements in adolescent-reported BDD severity on the BDD-YBOCS-SR with large effects from pre-treatment to post-treatment (*z* = -2.67, *p* = .008, *r* = .89), and pre-treatment to 2-month follow-up (*z* = -2.67, *p* = .008, *r* = .89), but no difference between post-treatment and follow-up (*z* = -0.41, *p* = .680, *r* = .14). Results for the BDD-YBOCS-PR followed a similar pattern, with significant differences from pre-treatment to post-treatment (*z* = -2.37, *p* = .018, *r* = .84) and follow-up (*z* = -2.37, *p* = .018, *r* = .89), but no difference between post-treatment and follow-up (*z* = -0.70, *p* = .482, *r* = .25).

#### Clinician-Rated Severity

 There were also significant improvements in CGI-Severity from pre-treatment to post-treatment (*z* = -2.23, *p* = .026, *r* = .74) and from pre-treatment to 2-month follow-up (*z* = -2.41, *p* = .016, *r* = .80), but there were no differences from post-treatment to 2-months follow-up (*z* = -0.58, *p* = .564, *r* = .19). On the CGI-Improvement scale, 22.2% were classified by independent raters as very much improved at post-treatment and 11.1% at follow-up; 44.4% were much improved at post-treatment and 55.6% at follow-up. At both post-treatment and follow-up, 22.2% were rated as minimally improved and one participant made no change (11.1%).

#### Insight

Wilcoxon tests also demonstrated that insight improved significantly from pre-treatment to post-treatment (*z* = -2.20, *p* = .028, *r* = .73) and from pre-treatment to 2-month follow-up (*z* = -2.32, *p* = .021, *r* = .74), but remained stable between post-treatment and 2-months follow-up (*z* = -0.17, *p* = .865, *r* = .06).

#### Functional Impairment

 Adolescent-reported functional impairment also improved from pre-treatment to post-treatment (z = -2.56, p = .010, r = .85), and from pre-treatment to 2-months follow-up (z = -2.67, p = .008, r = .89), with no differences found from post-treatment to follow-up (z = 0.32, p = .752, r = .11). The same pattern was observed for parentreported functional impairment, whereby there were significant differences between pre-treatment and post-treatment (z = -2.67, p = .008, r = .89), pre-treatment and follow-up (z = -2.67, p = .008, r = .89), but not posttreatment and follow-up (z = -1.90, p = .058, r = .63).

#### Comorbidity

Participants’ number of comorbid diagnoses on the MINI-Kid reduced significantly from pre-treatment to post-treatment (*z* = -2.00, *p* = .046, *r* = .67), and from pre-treatment and 2-month follow-up (*z* = -2.06, *p* = .039, *r* = .69). The difference between post-treatment and follow-up (*z* = -1.34, *p* = .180, *r* = .45) was non-significant. Wilcoxon pairwise tests showed that parent-reported anxiety did not reduce significantly from pre-treatment to post-treatment (*z* = -1.61, *p* = .108, *r* = .54), but there was a significant reduction from pre-treatment to 2-month follow-up (*z* = -2.43, *p* = .015, *r* = .81), and from post-treatment to 2-month follow-up (*z* = -2.53, *p* = .011, *r* = .84). Adolescent-reported depression reduced significantly from pre-treatment to post-treatment (*z* = -2.50, *p* = .012, *r* = .83), and from pre-treatment to follow-up (*z* = -2.38, *p* = .017, *r* = .79). Parent-reported depression also improved from pre- to post-treatment (*z* = -2.67, *p* = .008, *r* = .89), and from pre-treatment to follow-up (*z* = -2.67, *p* = .008, *r* = .89). Depression remained stable between post-treatment and follow-up based on adolescent-report (*z* = -0.63, *p* = .527, *r* = .21) or parent-report (*z* = -0.68, *p* = .495, *r* = .23).

## Discussion

CBT has been found to lead to symptom improvement for adolescents with BDD (Mataix-Cols et al., 2015), yet many adolescents do not have access to evidence-based interventions (Krebs et al., 2023). Even after receiving treatment, youth with BDD often remain symptomatic (Krebs et al., 2017). Hence, the present study aimed to evaluate the acceptability, feasibility, and preliminary efficacy of a telehealth-delivered, developmentally tailored CBT approach for treating adolescents with BDD. Modifications were made to current treatment protocols, including increased and standardised family involvement in therapy and additional modules targeting peer relationships and social media use. Motivational interviewing and attention training were also included as standardised components of the treatment.

**Table 3:** Means, standard deviations and time main effects for primary and secondary outcomes

	Treated Sample (*n* = 9)				Enrolled Sample (*n* = 16)			
	Pre-Treatment M (SD)	1-Week Post M (SD)	2-Month Post M (SD)	Statistic	Pre-Treatment M (SD)	1-Week Post M (SD)	2-Month Post M (SD)	Statistic
BDD-YBOCS-A	35.33^a^ (7.26)	24.33^b^ (14.51)	23.11^b^ (13.20)	χ^2^(2, *N =* 9) = 9.74, *p* = .008, *w* = 0.54	34.88^a^ (6.44)	28.69^b^ (12.30)	28.00^b^ (11.78)	χ^2^(2,*N* = 16) = 9.74, *p =* .008, *w* = 0.30
BDD-YBOCS-SR	29.22^a^ (7.63)	18.44^b^ (10.43)	18.22^b^ (10.44)	χ^2^(2,*N =* 9) = 15.25, *p <* .001, *w* = 0.70	28.44^a^ (6.54)	20.25^b^ (10.42)	20.13^b^ (10.45)	χ^2^(2,*N* = 16) = 14.56, *p <* .001, *w* = 0.46
BDD-YBOCS-PR	27.38^a^ (5.61)	18.11^b^ (10.49)	17.22^b^ (9.18)	χ^2^(2,*N =* 9) = 11.14, *p =* .004, *w* = 0.85	26.60^a^ (6.44)	21.44^b^ (9.87)	20.94^b^ (9.34)	χ^2^(2,*N* = 15) = 11.14, *p =* .004, *w* = 0.37
CGI Severity	5.33^a^ (1.00)	4.22^b^ (1.92)	4.11^b^ (1.76)	χ^2^(2,*N =* 9) = 11.22, *p =* .004, *w* = 0.62	5.38^a^ (0.95)	4.75^b^ (1.65)	4.69^b^ (1.58)	χ^2^(2,*N* = 16) = 11.22, *p =* .004, *w* = 0.35
Insight	16.56^a^ (4.98)	11.56^b^ (8.79)	12.00^b^ (7.76)	χ^2^(2,*N =* 9) = 6.07, *p* = .048, *w* = 0.34	15.56^a^ (4.41)	12.75^b^ (6.93)	13.00^b^ (6.20)	χ^2^(2,*N* = 16) = 6.07, *p =* .048, *w* = 0.19
Functional Impairment								
Adolescent Report	18.00^a^ (8.72)	11.78^b^ (10.99)	10.67^b^ (10.24)	χ^2^(2,*N =* 9) = 11.70, *p* = .003, *w* = 0.65	18.06^a^ (8.35)	14.56^b^ (10.21)	13.94^b^ (9.98)	χ^2^(2,*N* = 16) = 11.70, *p =* .003, *w* = 0.37
Parent Report	30.67^a^ (8.32)	15.56^b^ (10.78)	12.33^b^ (10.84)	χ^2^(2,*N =* 9) = 15.70, *p* < .001, *w* = 0.87	32.00^a^ (11.40)	23.50^b^ (15.46)	21.69^b^ (16.53)	χ^2^(2,*N* = 16) = 15.70, *p <* .001, *w* = 0.49
No. Comorbid Disorders	2.56^a^ (2.51)	2.11^b^ (2.62)	1.56^b^ (2.60)	χ^2^(2,*N =* 9) = 8.38, *p* = .015, *w* = 0.47	2.00^a^ (2.07)	1.75^b^ (2.08)	1.44^b^ (2.04)	χ^2^(2,*N* = 16) = 8.38, *p* = .015, *w* = 0.26
Anxiety								
Adolescent Report	29.67 (12.24)	22.56 (12.97)	21.44 (13.89)	χ^2^(2,*N =* 9) = 4.94, *p* = .085, *w* = 0.28	27.00 (10.77)	23.00 (10.79)	22.38 (11.42)	χ^2^(2,*N* = 16) = 4.94, *p* = .085, *w* = 0.15
Parent Report	20.22^a^ (8.58)	14.67^a^ (6.69)	11.89^b^ (6.31)	χ^2^(2,*N =* 9) = 10.80, *p* = .005, *w* = 0.60	20.81^a^ (7.17)	17.69^a^ (6.93)	16.13^b^ (7.58)	χ^2^(2,*N* = 16) = 10.80, *p* = .005, *w* = 0.34
Depression								
Adolescent Report	16.56^a^ (5.85)	9.67^b^ (8.88)	9.44^b^ (9.19)	χ^2^(2,*N =* 9) = 8.06, *p* = .018, *w* = 0.45	16.31^a^ (6.18)	12.44^b^ (8.52)	12.31^b^ (8.73)	χ^2^(2,*N* = 16) = 8.06, *p* = .018, *w* = 0.25
Parent Report	12.56^a^ (4.64)	6.11^b^ (4.59)	5.33^b^ (3.71)	χ^2^(2,*N =* 9) = 14.35, *p* < .001, *w* = 0.80	12.56^a^ (6.20)	8.94^b^ (7.01)	8.50^b^ (6.93)	χ^2^(2,*N* = 16) = 14.35, *p* < .001, *w* = 0.45

*Note: *Means with shared superscripts are not significantly different at 0.05; effect size w: > 0.10 = small effect; > 0.30 = medium effect; > 0.50 = large effect; CGI = Clinician’s Global Improvement; BDD-YBOCS-SR = Yale-Brown Obsessive Compulsive Scale modified for BDD – Self-Report; BDD-YBOCS-PR = Yale-Brown Obsessive Compulsive Scale modified for BDD – Parent-Report

Results of the study suggest the current treatment approach could lead to symptom improvement for some adolescents, as self-reported BDD symptoms remained stable throughout the baseline and generally improved following treatment for most participants. There were significant improvements in BDD severity observed at post-treatment, which were maintained at 2-month follow-up. The post-treatment response rate was 66.7% among the treated sample and 37.5% of the overall sample including dropouts. Moreover, significant improvements were found at post-treatment and 2-month follow-up for insight, functional impairment, depression, and the number of comorbid diagnoses. Parents, but not adolescents, also reported significant improvements in anxiety symptoms by 2-month follow-up. This could suggest that the treatment was not only associated with improvements in BDD symptomatology, but also better functioning, improved insight, and reductions in co-occurring psychopathology, particularly depressive symptoms. However, given secondary outcomes were uncontrolled at baseline, and several participants received other interventions between post-treatment and 2-month follow-up, these findings should be interpreted with caution. Participants and their parents who completed treatment also reported high satisfaction with the intervention.

While these results show the treatment may have promise, the current study also highlighted several difficulties involved in engaging and treating adolescents with BDD. Of the 22 participants who were initially offered treatment, only 16 were willing to participate. Among those who agreed to participate, seven dropped out early in treatment, with half of dropouts due to either parent or adolescent disengagement from the treatment. One participant also withdrew in the late stages of treatment. Among treatment completers, four participants at times refused to attend the sessions, and some reported difficulty attending treatment due to discomfort when discussing their BDD. Homework compliance was also relatively low, with participants, on average, only completing half or less than half of their assigned weekly homework. When compared to the low attrition rate in Mataix-Cols et al. (2015)’s trial of face-to-face CBT, the current study’s high dropout rate could suggest that for some young people with BDD, telehealth-delivery is not well tolerated. Indeed, some adolescents reported difficulty with the telehealth format, due to the video camera. When including participants who dropped out of treatment, the number of post-treatment responders (37.5%) was comparable to the number of responders in the Mataix-Cols et al. (2015) sample (40%). This suggests that the current treatment protocol did not lead to an overall improvement in outcomes for adolescents with BDD relative to previous trials.

Clinical implications can be drawn from the challenges identified in this study. Young people with BDD should be offered face-to-face treatment if this is their preference, where possible. When face-to-face cannot be accessed, therapists using telehealth may need to provide initial accommodations (e.g., video camera or self-view turned off), with the aim to gradually utilise the video camera as a tool in ERP over time. More time may need to be dedicated to rapport building, engaging in the young person’s interests, and exploring their broader goals, particularly with young people who are highly anxious about discussing their appearance concerns. Strategies to increase homework compliance may include automated email or SMS reminders between sessions. Future studies are needed to evaluate whether adolescents’ homework compliance differs between telehealth or face-to-face delivery. Moreover, co-design and qualitative interviews are much needed to understand adolescents’ preferences, to develop strategies to improve treatment engagement.

Single-case data in the current study suggest there is considerable variability in the response trajectories of adolescents’ BDD symptoms. Some participants made gradual steady change across the treatment, and some made very little change, while two participants (including one who withdrew from the study due to symptom improvement) made more rapid change early in treatment. The majority (*n* = 7 at post-treatment; *n* = 6 at follow-up) of the treated sample still met diagnostic criteria for BDD after receiving 18 h of CBT. Hence, future research is needed to understand predictors of response and remission. Drawing from single-case data, the two of the participants who achieved remission were both moderate in BDD severity at baseline. Higher BDD severity and lower insight at pre-treatment were also associated with less BDD symptom change at post-treatment. Higher baseline BDD severity was also associated less symptom change at 2-month follow-up, but there was no significant relationship between baseline insight and symptom change at this time point. Previous trials of CBT for adolescent BDD have failed to demonstrate consistent predictors of treatment response (Krebs et al. 2017; Rautio et al. 2022a, b), however in their uncontrolled study of 140 children and adolescents with BDD who received multi-modal treatment (CBT and medicationRautio et al. (2022a, b) found that baseline BDD severity did predict treatment outcomes at post-treatment, but not at 12-month follow-up. In this study, CBT was flexibly delivered over an average of 17 sessions of CBT (range = 2–80 sessions), and remission rates were more positive at 59% (Rautio et al. 2022a, b). Of note, 72% of the sample in this study were prescribed either an SSRI or anti-psychotic medication, whereas in the current study, only four participants were taking SSRIs before starting treatment. This suggests that some adolescents with BDD, potentially those with more severe symptoms, may require more sessions of CBT as well as medication. Brief telehealth-delivered CBT may offer an alternative to extended, face-to-face therapy within stepped-care models for adolescents with BDD. Future controlled studies exploring the dose-response relationship between CBT and treatment outcomes are needed.

Interestingly, most (*n* = 15) participants in the current study endorsed at least one Autistic trait on the MINI-Kid Autism screener and two had previous Autism diagnoses. In contrast, participants with Autism diagnoses were excluded in the Mataix-Cols et al. (2015) trial. The prevalence of Autistic traits in the current sample is unsurprising, given adolescent twins with BDD symptoms have been shown to have a fivefold increased likelihood of being identified as Autistic, compared to adolescents without BDD symptoms (Enander et al., 2018). Given the current study utilised a screener only, conclusions cannot be drawn about the true prevalence of Autism in the current sample. Hence, future research is needed using comprehensive diagnostic assessments, and to examine whether co-occurring Autism is a predictor of poorer response to CBT, as has been found in young people with OCD (Jassi et al., 2023).

### Limitations

Although multiple-baseline designs are useful for determining the feasibility of a treatment early in its development, and the current sample size was consistent with other multiple baseline studies, the sample was small. Secondary outcomes were also uncontrolled, limiting conclusions about these effects. Notably, recruitment of the small sample was slow, taking place over approximately 18 months. Similarly, in their larger study of 140 adolescents with BDD, Rautio et al. (2022a, b), enrolment took place over 5 years. This could suggest clinical trials for adolescent BDD are limited in terms of recruitment feasibility. However, these challenges must be balanced with the potential risk of neglecting an important area of research. Given individuals with BDD experience a 3-fold increased risk of death by suicide (Rautio et al., 2024), continued research efforts, including large RCTs with waitlist control conditions evaluating the efficacy of telehealth treatment approaches, are certainly warranted.

Because treatment was evaluated as an overall protocol, it is unclear whether the addition of specific treatment modules (e.g., peer relationship skills) improved outcomes over standard CBT. These modules were included based on literature that is largely cross-sectional, limiting conclusions about the direction of relationships between peer relationship factors, social media use, family functioning, executive functioning and BDD in adolescents. Longitudinal studies are much needed to provide further clarity and to assist with refining current treatments to appropriately target risk and maintenance factors. Future studies should also examine whether changes in factors such as family accommodation, peer relationship dynamics, and executive functioning occur following this modified CBT approach.

The sample was predominantly white, cis-gender girls, therefore future research is needed to test whether the treatment’s effectiveness generalises to boys and ethnic and gender minorities. As the families had volunteered for research, and many were already engaging with mental health professionals when referred to the study, they were potentially a more motivated and educated sample, limiting the generalisability of findings. The trial therapist rated their own adherence to the treatment protocol; a more optimal approach would have been to have independent raters evaluate treatment adherence. A last observation carried forward (LOCF) approach was used to manage missing data in the current study which has several limitations including the assumption of stability. As *p*-values were not adjusted, potentially inflating the type 1 error rate, conclusions should be interpreted with caution.

Adolescents, parents and post-treatment assessors were also aware of the purpose of the study and knew that a treatment assumed to be effective was being delivered. Further, pre-treatment diagnostic assessments and treatment sessions were conducted by the first author. Therefore, participants and assessors may have had a positive bias in their treatment outcome ratings. Several participants received other treatments between the post-treatment and 2-month follow-up assessments, which limits the validity of 2-month follow-up outcomes. Finally, treatment credibility and expectancy were not measured. Adult BDD research has found that higher treatment credibility is associated with a lower risk of dropout from remotely delivered BDD treatment (Bernstein et al., 2023), highlighting the need for future research in adolescent samples. Attrition was high, and those who dropped out early did not complete treatment satisfaction questionnaires, which limits conclusions about efficacy and acceptability. Qualitative interviews with stakeholders (i.e., young people with BDD and their families), which were not utilised in the current study, should also be conducted in future studies to help inform participant retention and outcomes.

## Conclusions

The current study suggests that telehealth-delivered developmentally tailored CBT may lead to symptom improvement for some adolescents with BDD who are willing to engage in therapy, however there is significant room for improvement. Future studies including large RCTs are needed to provide further support for this approach, as well as to test whether longer treatments and/or medication can enhance outcomes. Improvements to current therapy approaches are also needed to increase initial engagement in CBT, given many young people in this study dropped out early in treatment. Research into predictors of treatment response is also critically needed, to assist with the development of more effective treatments for adolescents suffering from BDD.

## Data Availability

Data can be accessed by contacting the research team directly.

## Declarations

### Clinical Trial Registration No

ACTRN12622000409774.
